# Promoting Health Literacy With Human-in-the-Loop Video Understandability Classification of YouTube Videos: Development and Evaluation Study

**DOI:** 10.2196/56080

**Published:** 2025-04-08

**Authors:** Xiao Liu, Anjana Susarla, Rema Padman

**Affiliations:** 1 Department of Information Systems W. P. Carey School of Business Arizona State University Tempe, AZ United States; 2 Accounting Information Systems Broad College of Business Michigan State University Lansing, MI United States; 3 Heinz College of Information Systems and Public Policy Carnegie Mellon University Pittsburgh, PA United States

**Keywords:** patient education, video analysis, video understandability, machine learning, cotraining, human-in-the-loop, augmented intelligence, artificial intelligence, AI

## Abstract

**Background:**

An estimated 93% of adults in the United States access the internet, with up to 80% looking for health information. However, only 12% of US adults are proficient enough in health literacy to interpret health information and make informed health care decisions meaningfully. With the vast amount of health information available in multimedia formats on social media platforms such as YouTube and Facebook, there is an urgent need and a unique opportunity to design an automated approach to curate online health information using multiple criteria to meet the health literacy needs of a diverse population.

**Objective:**

This study aimed to develop an automated approach to assessing the understandability of patient educational videos according to the Patient Education Materials Assessment Tool (PEMAT) guidelines and evaluating the impact of video understandability on viewer engagement. We also offer insights for content creators and health care organizations on how to improve engagement with these educational videos on user-generated content platforms.

**Methods:**

We developed a human-in-the-loop, augmented intelligence approach that explicitly focused on the human-algorithm interaction, combining PEMAT-based patient education constructs mapped to features extracted from the videos, annotations of the videos by domain experts, and cotraining methods from machine learning to assess the understandability of videos on diabetes and classify them. We further examined the impact of understandability on several dimensions of viewer engagement with the videos.

**Results:**

We collected 9873 YouTube videos on diabetes using search keywords extracted from a patient-oriented forum and reviewed by a medical expert. Our machine learning methods achieved a weighted precision of 0.84, a weighted recall of 0.79, and an *F*_1_-score of 0.81 in classifying video understandability and could effectively identify patient educational videos that medical experts would like to recommend for patients. Videos rated as highly understandable had an average higher view count (average treatment effect [ATE]=2.55; *P*<.001), like count (ATE=2.95; *P*<.001), and comment count (ATE=3.10; *P*<.001) than less understandable videos. In addition, in a user study, 4 medical experts recommended 72% (144/200) of the top 10 videos ranked by understandability compared to 40% (80/200) of the top 10 videos ranked by YouTube’s default algorithm for 20 ramdomly selected search keywords.

**Conclusions:**

We developed a human-in-the-loop, scalable algorithm to assess the understandability of health information on YouTube. Our method optimally combines expert input with algorithmic support, enhancing engagement and aiding medical experts in recommending educational content. This solution also guides health care organizations in creating effective patient education materials for underserved health topics.

## Introduction

### Research Background

Limited health literacy is a worldwide challenge [1]. The World Health Organization defines health literacy as “the cognitive and social skills which determine the motivation and ability of individuals to gain access, to understand, and use information in ways which promote and maintain good health.” It is estimated that almost 80% of the US adult population seek online health information [2]. However, only 12% of adults in the United States are considered proficient in their ability to meaningfully interpret health information [3]. Recently, a large-scale study assessing COVID-19–related health communications from state and federal agencies found that most information, including those from the US public health agency, the Centers for Disease Control and Prevention (CDC), exceeded recommended reading levels [4]. Health literacy is well recognized as a challenge for both individual and public health, with many adults lacking the requisite skills to engage successfully in the management of their health and health care [5]. Therefore, providing access to high-quality health information and patient education materials is essential for empowering patients, improving health and cost outcomes, and building societal resilience.

The internet has reduced much of the information asymmetry between health care providers and consumers by offering multiple avenues whereby patients can educate themselves with both user-generated and domain expert–generated content. However, for health consumers who search for health information on digital media platforms, health literacy divides can be exacerbated both by their own lack of knowledge and by algorithmic recommendations, with results that disproportionately impact minority groups and low health literacy populations [6]. Health consumers with higher health literacy levels seek online health information more frequently than those with lower health literacy levels [7] and report fewer difficulties in accessing high-quality, understandable health information.

Social media platforms such as YouTube (Google LLC), Instagram (Meta Platforms), and Facebook (Meta Platforms) have gained popularity among those searching for online health information due to the ease of posting and disseminating health information in multimedia format [8]. With patients regularly turning to social media platforms for health information and advice, pointing patients toward understandable and trustworthy video materials when needed is one mechanism to bridge the vast divide in health literacy and to enhance a patient’s ability to self-manage their medical conditions. While several patient education guidelines promote understandability and clear communication [9], particularly, it is not clear whether social media platforms are following such evidence-based guidelines for online information dissemination [10]. Health information from social media sources needs to be assessed on multiple criteria, such as understandability, accuracy and timeliness of the medical content, production quality, and credibility and trustworthiness of the content creators, among others.

The US Agency for Healthcare Research and Quality (AHRQ), a division of the federal Department of Health and Human Services, defines patient educational materials as understandable when consumers of diverse backgrounds and varying levels of health literacy can process and explain key health-related messages [9]. The Patient Education Materials Assessment Tool (PEMAT) is a systematic approach developed by AHRQ to evaluate and compare the understandability and actionability of patient educational materials in audiovisual format [9]. It is designed to be used by health care professionals to help determine whether patients will be able to understand and act on the information presented in the educational materials. PEMAT is the only guideline that includes a measure of objective assessment of audiovisual materials. Therefore, it has been widely adopted in evaluating patient educational materials in video format [11].

With the vast number of user-generated videos available on social media, there is both an urgent need and a unique opportunity to devise an automated approach to evaluate multimedia health information using multiple criteria. While patient educational guidelines such as PEMAT offer critical insights on how the materials should be evaluated for understandability and actionability, relying on health care professionals to manually annotate all the videos is not sustainable or scalable.

### Research Objective

In this study, we aimed to address these gaps via 2 main objectives. First, we developed a human-in-the-loop augmented intelligence approach to assess the understandability of patient educational videos deployed on the YouTube platform. We gathered a diverse range of diabetes-related educational videos from YouTube, leveraging its position as the largest video-sharing platform, to create a research test bed. Our second aim was to highlight the importance of understandability for various stakeholders, including content creators and health care providers, as defined by the PEMAT guidelines. Extracting specific multimedia features from these videos, we performed a computational evaluation of understandability in accordance with the PEMAT guidelines for audiovisual materials developed by AHRQ. The selected features were aligned with the PEMAT criteria, and we used a cotraining classification method that incorporated human feedback to ensure accurate assessments. We evaluated the impact of video understandability on viewer engagement, offering insights for content creators on how to improve their educational videos. In addition, we conducted a user study to examine the perceived effectiveness of understandable content from the perspective of health care professionals.

### Related Work

#### Evaluating Online Health Information in Promoting Health Literacy

In this section, we review recent studies that evaluate online health information and their suitability for promoting health literacy based on existing guidelines. To summarize the related literature, we developed a taxonomy in Table 1 that specifically focused on the type of health information, the guidelines or assessment tools used, the criteria used in the evaluation, and the key findings.

**Table 1:** Summary of studies evaluating online health information.

Study	Data	Assessment tool	Focus	Finding
Kang and Lee [12], 2019	A total of 85 videos from hospital websites	PEMAT^a^	Understandability, actionability, and usefulness	The average understandability rating was 49.5%, and the actionability rating was 31.4%. The average usefulness score was 4.3 on a 7-point scale.
McClure et al [13], 2016	A total of 9 print and 4 online patient education materials	SMOG^b^, PMOSE^/^IKIRSCH^c^, PEMAT, and CDC^d^ Clear Communication Index	The reading level of the publicly available patient education materials	Reading levels of available patient education materials exceed the documented average literacy level of the US adult population.
Johnson et al [14], 2020	A total of 4 text-based patient education materials for sickle cell disease	The Flesch Reading Ease Formula, the Flesch-Kincaid Reading Tool, SMOG Readability Formula, the PEMAT, CDC Clear Communication Index, and PMOSE/IKIRSCH tool	Readability, grade level, understandability, and actionability	Literacy levels of the patient education materials were higher than the recommended standards.
Rooney et al [15], 2020	A total of 54 patient education materials from high-performing neurosurgery hospitals and professional societies	Six readability indices	Readability	Publicly available online patient educational materials for stereotactic radiosurgery were written at reading levels above the national recommendation. Furthermore, many lacked information identified as important by patients.
Williams et al [16], 2016	A total of 950 written patient educational materials	Three guidelines from the AMA^e^, CDC, and NIH^f^ for written materials	Readability, structure, and presentation	Materials were consistently written at a readability level that was poorly suited for patients with low health literacy.
Kunze et al [17], 2020	A total of 50 YouTube videos of meniscus	JAMA^g^ benchmark criteria and Global Quality Score	Quality and reliability	Information on the meniscus found in YouTube videos was of low quality and reliability.
Sanderson et al [18], 2016	One YouTube video about genome sequencing	—^h^	Understandability and knowledge increased	A total of 79% reported that the video was easy to understand, satisfaction scores were high, and knowledge increased significantly.
Salama et al [19], 2020	A total of 53 YouTube videos about hypospadias	PEMAT	Understandability and actionability	Only 5.6% of videos were understandable, and 15.1% were actionable. The vast majority of hypospadias-related YouTube content was not appropriate for users with low health literacy.
Desai et al [20], 2013	A total of 607 videos from Mayo Clinic’s social media health network	SAM^i^	Suitability and user engagement	Health care organizations produce very few videos with high SAM scores. An optimal video is no more likely to engage users than less optimal videos.

^a^PEMAT: Patient Education Materials Assessment Tool.

^b^SMOG: Simple Measure of Gobbledygook.

^c^PMOSE/IKIRSCH: the Peter Mosenthal and Irwin Kirsch readability formula.

^d^CDC: Centers for Disease Control and Prevention.

^e^AMA: American Medical Association.

^f^NIH: National Institutes of Health.

^g^JAMA: Journal of The American Medical Association.

^h^Not applicable.

^i^SAM: Suitability Assessment of Materials.

Studies have examined online patient educational materials ranging from those created by professionals, such as hospitals and health systems, to user-generated content by the layperson [15,17]. Several studies have focused on health information in text format [12-14,16], while others focused on video content [17-19]. Evaluation guidelines used in prior studies include the Clear Communication Index from the CDC [14], PEMAT from AHRQ [19], Benchmark criteria from the *Journal of the American Medical Association* [17], Suitability Assessment of Materials [20], Global Quality Score [17], and readability indices [15]. Readability, content organization, and presentation are critical to health care consumers. These factors impact how patients consume educational materials and whether the medical information can be delivered effectively. A host of studies assessing these topics suggest that most education materials may be too complicated for patients to comprehend [5,21], especially for those with low health literacy [22].

Prior studies have relied on the judgment of domain experts, such as health professionals, to evaluate online health information. Content rated by an expert (such as medical or health professional staff) is the most common approach to assessing videos focused on health education. Health and medical websites are increasingly encouraged to apply for quality certificate assessments as proof of evidence that they are reliable sources of information [23]. However, as the volume of online health information grows exponentially, using experts to evaluate content is not a scalable solution.

#### Augmented Intelligence and Human-in-the-Loop Training Methods

Evaluation of health care video content requires domain expertise. Given the amount of user-generated video content available, it is not feasible to generate a large, labeled dataset for typical stand-alone machine learning (ML) and natural language processing (NLP) methods. Due to the high level of uncertainty and criticality in health care and problem diversity, our objective was to introduce humanlike cognitive capabilities into artificial intelligence (AI) systems to develop an augmented intelligence approach. While AI, ML and other automation technologies have make substantial advances in recent years, many important health care problems are often solved through the collaboration of human beings and machines [24-27]. Human-in-the-loop augmented intelligence is defined as an intelligent model that requires human interaction. Bott et al [28] used human-in-the-loop software design to develop a conversational agent to support nurse teams in mitigating risks of hospitalization. Wang et al [27] used a human-in-the-loop method to predict suicidal ideation. In this type of intelligence system, the human is always part of the system and consequently influences the outcome in such a way that the human gives further judgment if a low-confidence result is given by an algorithm. This approach readily allows us to address problems and requirements that may not be easily trained or classified by ML.

Cotraining is a multiview learning paradigm that exploits unlabeled data in addition to labeled data to improve learning performance [29]. In ML, unlabeled data are often substantially cheaper and more plentiful than labeled data. YouTube contains thousands of health care–related videos. However, annotating these videos requires significant human effort. Given the domain expertise required, it is not feasible to obtain a large amount of annotated video data. Cotraining trains 2 learners from 2 different views and lets the learners label the most confident unlabeled instances to enlarge the training set of the other learner [30]. When the 2 learners are inconsistent, a human expert will evaluate the performance and decide on the label. Such a process is repeated until a stopping condition is met. Intuitively, each example contains 2 “views,” and each view contains sufficient information to determine the label of the example. This redundancy implies an underlying structure of the unlabeled data (because they need to be “consistent”), and this structure makes the unlabeled data informative. This approach has been used for a variety of learning problems, including recommender systems [31], text classification [32], NLP [33], and image recognition [32]. The cotraining process is viewed as a combinative label propagation over 2 views. Obtaining labels can be expensive or time consuming because of the involvement of human experts in this research context. Most learning tasks can be made more efficient in terms of labeling cost by intelligently integrating specific unlabeled instances to be labeled by experts.

### Research Question

Despite the growing attention of policy makers and health care providers, it is evident that health educational materials remain too complicated for patients to comprehend [4]. Evaluation of online health information is an urgent issue and is amplified when considering that 80% of Americans search for health information online, and only 12% have proficient health literacy to correctly interpret and use it. In this study, we seek to address the following research question: How can we design a scalable, generalizable, and sustainable approach to combine human cognitive power with ML and NLP methods to evaluate the understandability of user-generated video content for patient education and health literacy promotion?

## Methods

### Overview

**Figure 1:**
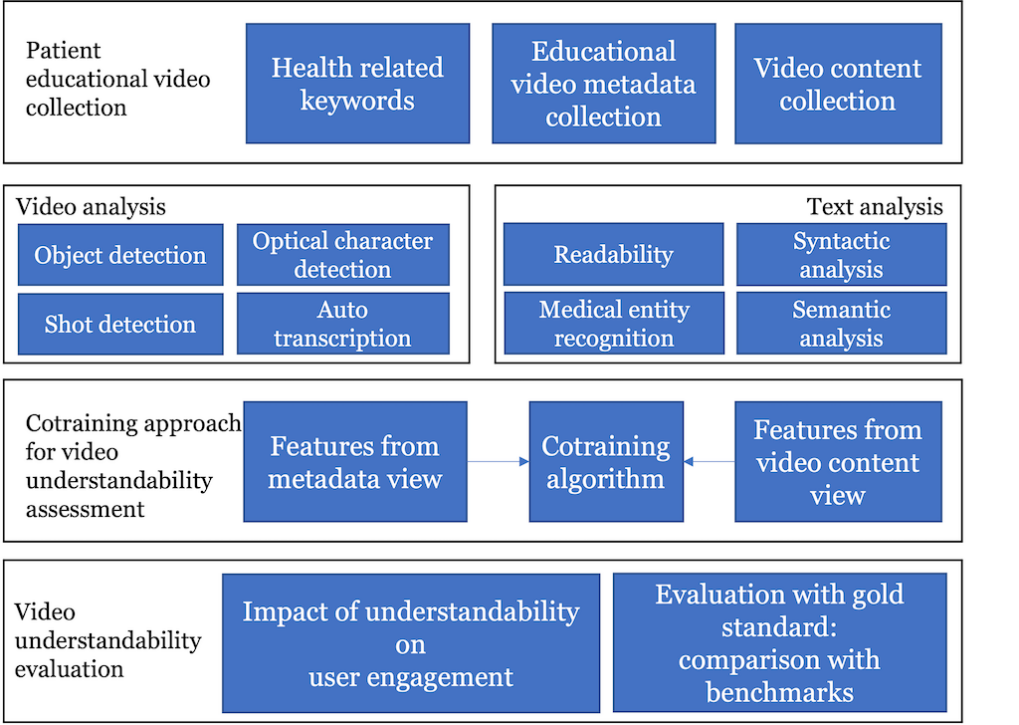
The research framework to assess health care video understandability.

We propose a scalable, generalizable, and augmented-intelligence-based cotraining approach to assess the understandability of YouTube videos for patient education. Our method can be used outside patient education in the broader context of understandability of health information. Figure 1 illustrates our approach, which consists of 5 components: video collection, video analysis, text analysis, cotraining approach for video understandability assessment, and understandability evaluation.

### Research Context

YouTube, the largest video-sharing social media platform, hosts >100 million videos providing information on the pathogenesis, diagnosis, treatments, and prevention of various medical conditions [34]. Patient educational materials in visual format may be easier to comprehend and adhere to. For patients who need complex medical information, health care advice in a video format may make it more convenient, understandable, and actionable and improve the outcomes and efficiency of care. While this plethora of user-generated content can be leveraged by patients to improve health literacy and adherence to treatments, criticisms of social media use in health care have also raised serious concerns [35]. Our study can offer a path toward patient education and empowerment and improved health literacy of the population by providing clinicians and patients with the ability to easily retrieve understandable and relevant video-based health education content.

This study focuses on the content assessment for diabetes, as it is among the most prevalent chronic condition in the United States and many other parts of the world. More than 100 million US adults are now living with diabetes or prediabetes, according to the CDC 2020 [36]. Diabetes is a contributing factor to many other serious health conditions, such as heart disease, stroke, nerve and kidney diseases, and vision loss [36-38]. To reduce the impact of prediabetes and type 2 diabetes, health care institutions and medical professionals are applying a multipronged approach to increase awareness of diabetes and its consequences and promote patient education on self-management and lifestyle behavior change programs to improve healthy eating habits and increase physical activity [39]. Clear, multimedia-rich, and trustworthy videos can complement and support clinician and public health efforts.

### Video Collection

On the basis of inputs from health care professionals and patient searches on diabetes-related discussion forums, we identified 235 search keywords related to the education of patients with diabetes. These keywords covered various aspects of the education of patients with diabetes, including general information about the disease, treatments, laboratory tests, prevention, self-management procedures, and lifestyle management. These keywords are available in Multimedia Appendix 1. We collected the top 50 videos for each search term using the YouTube data application programming interface (API) and stored the video IDs, their rankings, and metadata in a database for further analysis. The attributes we collected about each video from YouTube data API are available in Multimedia Appendix 2. Attributes related to video snippets and content details were generated at the time of video upload, while video use was generated by user engagement over time and the statistics were from the day of video data collection. After we collected video metadata from YouTube API, we used the stored video IDs and YouTube-DL, a command-line program, to download video content (ie, mp4 files) from YouTube. In total, we collected 9873 unique videos using 235 search terms, which served as the data for this study on video understandability.

YouTube offers a diverse range of content and perspectives, featuring contributions from health care professionals, patients, caregivers, and the general public. Our dataset included professionally produced videos by health care organizations and individuals on the basics of diabetes, its complications, and treatments. It also contained research presentations from renowned researchers and medical experts on the latest research developments and scientific findings about the disease. However, low-quality content or inaccurate videos produced by both individuals and organizations also contributed to the diversity, which presented a challenge for assessing video understandability.

### Main Outcome Variable: Video Understandability

To obtain the ground truth for our outcome variable, video understandability, we relied on experts’ consensus perspective based on the PEMAT for audio and video materials [9], as it is the only systematic method developed to assess video content. Table 2 lists our adaptation of PEMAT, which focuses on 4 aspects of video materials, specifically: content, word choice and style, organization, and layout and design, with multiple criteria within each aspect. The understandability score of a video was calculated based on the scores for each criterion with the following equation. When a video was scored >50%, it was considered to have high understandability.



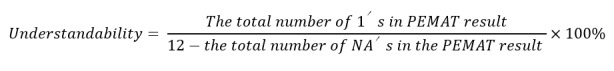



**(1)**

**Figure 2:**
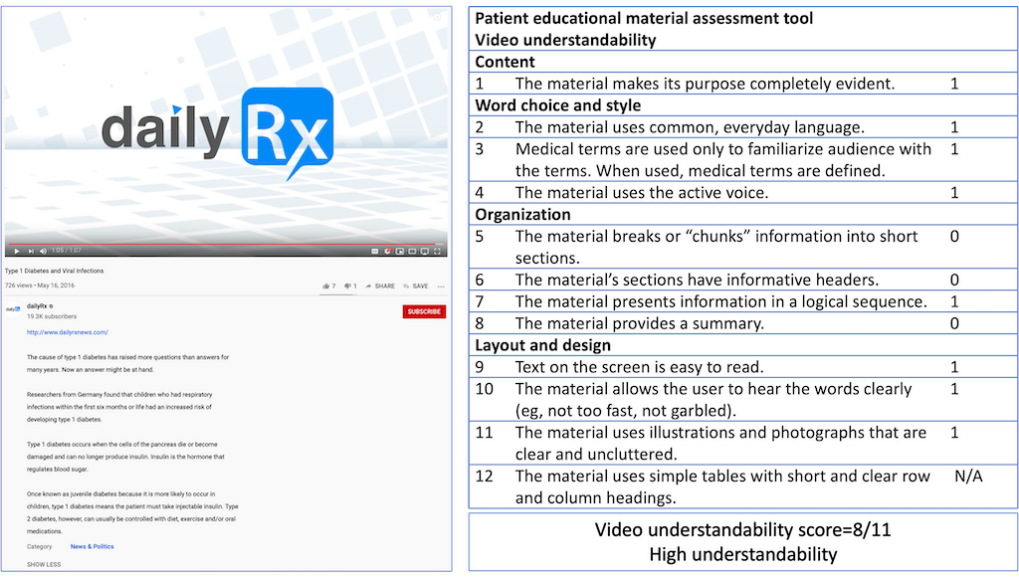
An illustrative example of expert annotation on video understandability.

Given the volume and scope of health care videos on YouTube, manual evaluation as well as annotation of a large number of videos by domain experts can be time consuming and costly, hence impractical. Our approach used a semisupervised method called cotraining, which not only learns from the labeled observations but also leverages the unlabeled instances to improve model performance. A total of 600 diabetes-related videos were randomly selected from our corpus of 9873 unique videos as the initial labeled dataset for cotraining. Another 100 videos were sampled for evaluation. Sample size calculation indicated that <500 videos were needed to achieve high interrater reliability (κ>0.80) with multiple raters [40]. The remaining videos were used as unlabeled data to evaluate the effectiveness of cotraining for semisupervision. When the ML models yielded inconsistent results, the medical experts reviewed the videos and provided supervision, according to PEMAT. Four physicians, trained to use these guidelines, labeled these videos for video understandability according to the PEMAT guidelines in Table 2. They watched a video; assessed the video according to the criteria within content, word choice and style, organization, layout, and design; and assigned them 0, 1, or not applicable (N/A). Figure 2 demonstrates the expert evaluation measures and results. Four domain experts watch a videoo [41] and assess the video according to its content, word choice and style, organization, and layout and design. They assign scores from 0, 1, or N/A to items Table 2. The video in Figure 2 is considered to have high understandability.

The PEMAT is designed to be completed by health care professionals, including health care providers, health librarians, and other clinical practitioners. The selected raters fall into the targeted user group who are qualified to use the PEMAT tool to rate the videos. Before they started working on annotation, all of them carefully studied the PEMAT user guide [42]. To maximize the consistency among these raters, we had each rater independently rate the same 10 videos. A study session was held with these 4 raters to discuss items with discrepancies. Each rater provided their rationale for the rating provided. The group reviewed the PEMAT user guide to clarify how each item was intended to be rated and come to a consensus. Then, they rated the rest of the videos based on the consensus. We used the intraclass correlation coefficient [43] to assess the interrater reliability of the annotation at the video level. To ensure there is an agreement on every video, a fifth rater reviewed and consolidated the videos with discrepancies. Each video took approximately 10 minutes to review. The interrater reliability of the video understandability score was 87%. Table 3 summarizes video understandability scores (according to the PEMAT guidelines).

**Table 2:** Patient educational material assessment tool (video understandability) for audio and video materials.

Content		Score^a^
	The material makes its purpose completely evident.	0, 1
**Word choice and style**		
	The material uses common, everyday language.	0, 1
	Medical terms are used only to familiarize the audience with the terms. When used, medical terms are defined.	0, 1
	The material uses the active voice.	0, 1
**Organization**		
	The material breaks or “chunks” information into short sections.	0, 1, N/A^b^
	The material’s sections have informative headers.	0, 1, N/A
	The material presents information in a logical sequence.	0, 1
	The material provides a summary.	0, 1, N/A
**Layout and design**		
	The text on the screen is easy to read.	0, 1, N/A
	The material allows the user to hear the words clearly (eg, not too fast, not garbled).	0, 1, N/A
	The material uses illustrations and photographs that are clear and uncluttered.	0, 1, N/A
	The material uses simple tables with short and clear row and column headings.	0, 1, N/A

^a^Scoring: 0=disagree, 1=agree.

^b^N/A: not applicable.

**Table 3:** Video understandability annotation (on a binary scale; N=700).

Variables	Number of “0” scores (no), n (%)	Number of “1” scores (yes), n (%)	Number of N/A^a^, n (%)
The material makes its purpose completely evident.	175 (25)	525 (75)	0 (0)
The material uses common, everyday language.	183 (26.1)	517 (73.9)	0 (0)
Medical terms are used only to familiarize the audience with the terms. When used, medical terms are defined.	241 (34.4)	459 (65.6)	0 (0)
The material uses the active voice.	174 (24.9)	526 (75.1)	0 (0)
The material breaks or “chunks” information into short sections.	548 (78.3)	143 (20.4)	9 (1.3)
The material’s sections have informative headers.	601 (85.9)	90 (12.9)	9 (1.3)
The material presents information in a logical sequence.	164 (23.4)	536 (76.6)	0 (0)
The material provides a summary.	458 (65.4)	233 (33.3)	9 (1.3)
The text on the screen is easy to read.	137 (19.6)	294 (42)	269 (38.4)
The material allows the user to hear the words clearly.	97 (13.9)	539 (77)	64 (9.1)
The material uses illustrations and photographs that are clear and uncluttered.	111 (15.9)	338 (48.3)	251 (35.9)
The material uses simple tables with short and clear row and column headings.	192 (27.4)	57 (8.1)	451 (64.4)
Understandability	315 (45)	385 (55)	0 (0)

^a^N/A: not applicable.

### Data Analysis

Video data analysis forms the building blocks for designing our ML approach for evaluating patient educational videos. In processing the video data, we extracted the features according to PEMAT criteria in Table 2 with video analysis techniques from the Google Cloud platform. Table 4 below summarizes the features we extracted from video data processing results.

The PEMAT guidelines suggests that breaking the information into small chunks or sections is positively related to video understandability. We used scene detection methods to detect the number of scenes in a video as an indicator of whether the videos were organized in small sections. We built on prior work that defined a scene as 1 of the subdivisions of a movie or a play, in which the setting is fixed or when it presents continuous action in 1 place [44]. A scene comprises a single, complete, and unified event or segment of a movie. A scene normally occurs in 1 location and deals with 1 action; the end of a scene is often indicated by a change in time, action, or location. Scene detection is a widely adopted method in computer vision and video analytics for video classification, video understanding, and management [45]. Video content analysis relies on scene detection to extract story units and segments. Scene change detection estimates the subsections in a video [46]. Scene change detection is important in a number of video applications, including video indexing and semantic feature extraction. Video content analysis relies on scene detection to extract story units and segments.

A video transcript is a text version of a video’s audio track. Video transcription techniques can extract video narratives, which convey a significant portion of the information in the videos [47]. The quality of narratives also may affect viewers’ understanding of the videos. PEMAT evaluates if the material allows the user to hear the words clearly. We conduct video transcription to perform in-depth content analysis and assess the clarity of narratives. Optical character recognition (OCR) is used to detect and extract text, tables, or illustrations in the videos. Layout and design are an important aspect of patient education video evaluation. Text on the screen should be easy to read. The illustrations and tables should have clear headings. OCR can extract features related to the clarity of text, tables, and illustrations and enable the evaluation of video layout and design.

PEMAT expects a video or multimedia material with narration to allow the viewer to hear the words clearly. The narrator or voiceover should speak at a clear and moderate pace, avoiding speech that is too fast, garbled, or difficult to understand. The video transcription algorithm returns not only the transcript but also the confidence score of the predicted transcript. The confidence score reflects whether the speech is clear. We also assessed whether the text on the screen was easy to read with OCR. Audiovisual materials that were overcrowded with words or had text that flashed briefly on the screen were difficult to read and understand. This item was N/A if no text appeared in the material or a narrator read all the text out loud because the material did not rely on the viewer to read the text. We used OCR to detect text in the videos and the confidence score of OCR as a proxy for whether the text was easy to recognize.

**Table 4:** Features from the Google Cloud Video Intelligence application programming interface.

Tasks and features		Description
**Detect shot changes**		
	Number of scenes in a video	The total number of scenes throughout the video
**Optical character recognition**		
	Text on screen	A string of text detected in the video
	Text confidence score	The confidence score of a detected text
**Video transcription**		
	Transcribed text	The automated video transcription results
	Transcription confidence score	The confidence score of a transcribed text

### Text Analysis for Videos

#### Readability

PEMAT requires that the material should use common, everyday language that would be easy to understand for most consumers or patients nearly all the time. To assess this criterion, we conducted a readability analysis and used the Flesch-Kincaid readability test to determine how easily the video’s description and transcription could be understood. The Flesch-Kincaid readability test was developed under contract to the US Navy research in 1975 [48]. It has been widely adopted, especially in the public health domain, to assess how easy it is to read a given material [49,50].

#### Syntactic Analysis

PEMAT assesses whether the material uses active voice. It is often argued that the passive voice will result in a structure that is more verbose than the active voice and, therefore, harder to understand and that the meaning of the passive voice is indirect and less forceful than the active voice [51]. Therefore, the use of active voice is highly advocated in patient educational materials, medical writings, and related areas. According to PEMAT, if the material overall uses active voice, this criterion is met. To automatically assess this criterion, we used part-of-speech tagging, a common linguistic technique, to detect the category of verbs in the video description and narratives and compute the number of verbs in active voice. The number of verbs in active voice was extracted with part-of-speech tagging. The verbs in active voice belong to the following tag set: verb, base form, verb, past tense, verb, gerund or present participle, verb non-3rd person singular present, and verb, 3rd person singular present.

#### Medical Entity Recognition

We adopted a bidirectional long short-term memory model from prior work to extract 6 types of medical terms from the text data [52]. Table 5 lists the medical term categories and provides explanations. These 6 categories cover most medical terminologies used in patient educational materials and communications [53].

A total of 5000 sentences were randomly selected from the video description and transcription test bed, with 4000 in the training set and 1000 in the test set. Two expert annotators independently labeled the sentences for semantic types. We used Cohen κ to measure interannotator reliability. The κ value was 0.90 for the medical terminology annotation. A third annotator reviewed the disagreements and made the final judgments. Finally, the ground truth was generated, containing 4000 training sentences and 1000 test sentences. The statistics of the training and test sets are shown in Table 6.

We trained an embedding model using the skip-gram method in Word2vec and implemented a bidirectional long short-term memory model to extract medical terms from video descriptions and transcriptions at the sentence level. Overall, the model achieved a precision of 87.4%, a recall of 87.8%, and an *F*_1_-score of 87.3%. We also conducted several experiments to evaluate the classification performance of our method in comparison to dictionary-based approaches and state-of-the-art methods, such as conditional random fields. Performance is reported in Multimedia Appendix 3. We then extracted medical terms from video descriptions and transcriptions using the model.

**Table 5:** Medical terminologies used in patient educational materials and communications.

Medical term category	UMLS^a^ semantic type	Examples
Body part	bdsy (body system), blor (body location or region), and bpoc (body part, organ, or organ component)	Liver, foot, and pancreas
Chemicals or drugs	chem (chemical), chvf (chemical viewed functionally), chvs (chemical viewed structurally), clnd (clinical drug), elii (element, ion, or isotope), enzy (enzyme), hops (hazardous or poisonous substance), inch (inorganic chemical), orch (organic chemical), and phsu (pharmacologic substance)	Insulin, metformin, and lantus
Medical devices	drdd (drug delivery device) and medd (medical device)	Insulin pen and glucometer
Medical events	acab (acquired abnormality), dsyn (disease or syndrome), inpo (injury or poisoning), mobd (mental or behavioral dysfunction), patf (pathologic function), and sosy (sign or symptom)	Nausea, ketosis, and diabetes
Medical professionals	humn (human) and famg (family group)	Physician, diabetes educators, and nurses
Medical procedures	lbpr (laboratory procedure), lbtr (laboratory or test result), and topp (therapeutic or preventive procedure)	HbA_1c_ and creatinine

^a^UMLS: Unified Medical Language System.

**Table 6:** Statistics of the training and test sets.

	Training set	Test set
Number of sentences	4000	1000
Number of mentions of body part	227	101
Number of mentions of chemicals and drugs	2181	538
Number of mentions of medical devices	545	126
Number of mentions of medical events	784	245
Number of mentions of medical professionals	67	18
Number of mentions of medical procedures	197	53

#### Semantic Analysis

Multiple PEMAT criteria were evaluated using semantic analysis methods. First, PEMAT expects materials to have a summary of the key points or a review of the key points of the material, either in writing or orally. The summary usually comes at the end of the material and starts with summary words. Therefore, we curated a comprehensive list of summary words and phrases from multiple sources and used them to detect whether a material provided a summary.

Second, PEMAT suggests that information in the material should be presented in an order that makes sense to the user. Main messages or the most important ideas should be at the beginning of sections or in bulleted lists because users tend to pay more attention to them [13]. To measure whether the material presented information in a logical sequence, we evaluated the use of transitional words and phrases in the material. A transition is a change from one idea to another in writing or speaking and can be achieved using transition terms or phrases, which are most often placed at the beginning of sentences, independent clauses, and paragraphs, to create a clear connection between ideas or groups of ideas. Transitions are used to create “flow” in writing or speaking and make its logical development clearer to the audience. The use of transition words and phrases can improve the logical connections in writing and speech [54]. Transition words and phrases can be grouped into categories such as causation, chronology, combinations, contrast, example, clarification, summary, and more. We collected common transitional terms and phrases under these categories as a proxy to measure whether the material presents information in a logical sequence. Textbox 1 [55,56] lists all the words and phrases used to identify transitions and summaries.

Third, we evaluated whether the material makes its purpose evident. According to the PEMAT user guide, this criterion refers to whether the material uses a title or upfront text that tells the reader what the material is about. We implemented this criterion by checking whether each video had a title, tags, and description. YouTube suggests that tags are descriptive keywords that content creators can add to the video to help viewers find the content. The video’s title, tags, and description are important pieces of metadata for the video’s discovery and should provide critical information about the purpose of the video so that viewers can find the video and decide whether to watch it.

**Words and phrases for summary and transition.**

- Summary or conclusion: Finally, in a word, in brief, briefly, in conclusion, in the end, in the final analysis, on the whole, thus, to conclude, to summarize, in sum, to sum up, in summary, lastly, in short, by and large, consequently, as a result, hence, overall, in conclusion, and after all
- Transition: Accordingly, as a result, and so, because, consequently, for that reason, hence, on account of, since, therefore, thus, after, afterwards, always, at length, during, earlier, following, immediately, in the meantime, later, never, next, once, simultaneously, so far, sometimes, soon, subsequently, then, this time, until now, when, whenever, while, additionally, again, also, and, or, not, besides, even more, finally, first, firstly, further, furthermore, in addition, in the first place, in the second place, last, lastly, moreover, next, second, secondly, after all, although, and yet, at the same time, but, despite, however, in contrast, nevertheless, notwithstanding, on the contrary, on the other hand, otherwise, thought, yet, as an illustration, eg, for example, for instance, specifically, to demonstrate, to illustrate, briefly, critically, foundationally, more importantly, of less importance, primarily, above, centrally, opposite to, adjacent to, below, peripherally, below, nearby, beyond, in similar fashion, in the same way, likewise, in like manner, ie, in other word, that is, to clarify, to explain, in fact, of course, undoubtedly, without doubt, surely, indeed, for this purpose, so that, to this end, in order that, and to that end

### Cotraining Approach for Video Understandability Assessment

#### Overview

We defined the video understandability classification in the context of patient education as a multiview learning and binary classification problem. Due to the vast amount of user-generated videos available and the cost to annotate the videos manually, it is essential to deploy an augmented intelligence approach in this context. The cotraining approach enabled us to accomplish this task with limited human effort and incorporate domain experts’ assessment when the results from ML models were insufficient for unambiguous classification. Our dataset included video metadata and video content data. We developed classifiers from 2 sufficient and conditionally independent views (ie, video metadata and video content) to assess video understandability. Following a feature-based design, we engineered the features based on the evaluation criteria in the PEMAT guideline. The classification model we used in the cotraining approach was logistic regression due to its high interpretability, which can benefit health care organizations and health content creators in their future content creation process.

#### Cotraining-Based Understandability Classification

**Figure 3:**
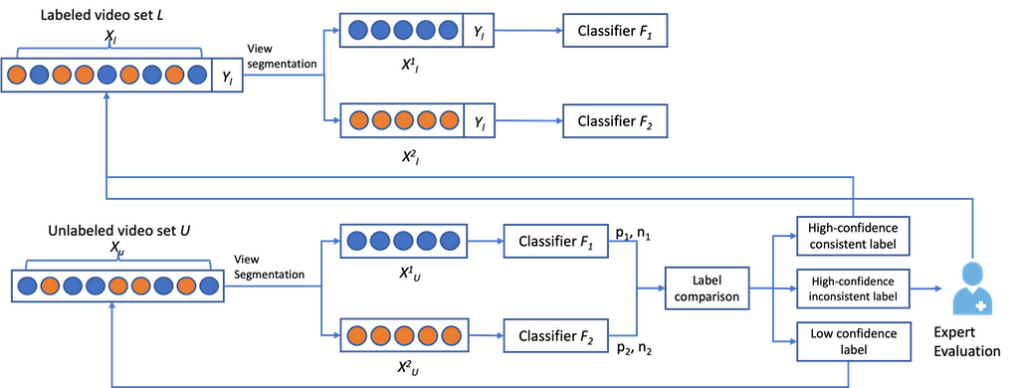
A human-in-the-loop cotraining approach to assessing video understandability.

Figure 3 illustrates the steps in the cotraining approach for video understandability classification. This consists of the following components: a set of *L*-labeled videos and a set of *U*-unlabeled videos, a classifier *F_1_* trained with features from video metadata view, a classifier *F_2_* trained with features from video content view, and a hyperparameter confidence threshold. The video metadata contains the video title, video description, video tags, and video use information. It represents how the content creators would like the viewers to perceive the video. The video content view captures the information delivered by the video. Combining video content and video metadata gives us a comprehensive view of the videos on YouTube. An initial labeled dataset *L* and an unlabeled dataset *U* are given. The cotraining process in this study is presented in Textbox 2.

**Pseudocode for the cotraining algorithm.**

**Input**

- A set *L* of labeled video examples
- A set *U* of unlabeled video examples

**Output**

- A set *L′* of labeled video examples

**Procedures**

- Loop for K iterations

1. Train a classifier *F_1_* on *L* that considers only video metadata features and train a classifier *F_2_* on *L* that considers only features from video content features
2. Use the trained classifiers to make predictions on videos in *U*
3. Extract p1 positive and n1 negative examples from *U* on which F1 has the most confident predictions, specified by a predefined confidence threshold
4. Extract p2 positive and n2 negative examples from *U* on which F2 has the most confident predictions, specified by a predefined confidence threshold
5. Compare p1 with p2 and n1 with n2
6. If a video appears in both p1 and p2 or in both n1 and n2, move the video and its label from *U* to L’
7. If a video appears in both p1 and n2 or both p2 and n1, expert reviewers annotate inconsistent labels and add the final label of this video to L’
8. Halt when U is empty or no new videos are added to the labeled set L’

#### Features From Video Metadata View

In the video metadata view classifier, we leveraged the features generated from video metadata to classify video understandability. As described earlier, each video’s metadata contains the video title, description, and tags, which are submitted by the content creator, suggesting the purpose of a given video. A video with good understandability for patients uses common daily language, so text preprocessing techniques are used to identify the total number of words, sentences, and unique words from the *video description*. A bidirectional long short-term memory named entity recognition model is used to extract the number of medical terms, of which those in the *video description* are included in the metadata view classification. Table 7 summarizes the features we extracted from the video metadata view, the methods used to derive the measure, and the PEMAT criteria they fall under.

**Table 7:** Features for video understandability classification from the video metadata view.

Name	Description	Method	PEMAT^a^ criterion
Has title	Whether the video has a title	Metadata collection	The material makes its purpose evident
Has description	Whether the video has a text description	Metadata collection	The material makes its purpose evident
Has tags	Whether the video has tags	Metadata collection	The material makes its purpose evident
Description readability	The automated readability index of the video description	Readability analysis	The material uses common everyday language
Active word count	The number of verbs in active voice in the video description	Syntactic analysis	The material uses active voice
Summary word count	The number of summary words in the video description	Semantic analysis	The material provides a summary
Transition word count	The number of transition words in the video description	Semantic analysis	The material presents information in a logical sequence
Video duration	The total length of the video in seconds	Metadata collection	Medical information encoded in the video metadata
Description word count	The total number of words in the video description	Metadata collection	Medical information encoded in the video metadata
Sentence count	The total number of sentences in the video description	Metadata collection	Medical information encoded in the video metadata
Description unique words	The total number of unique words in the video description	Metadata collection	Medical information encoded in the video metadata
Description medical term count	The total number of medical terms in the video description	Medical entity recognition	Medical information encoded in the video metadata

^a^PEMAT: Patient Education Materials Assessment Tool.

#### Features From Video Content View

In the video content view, we derived features from the video narratives, video shots, and associated confidence scores. We generated a narrative readability score to examine whether the narrative material uses common everyday language. Part-of-speech tagging was used to extract verbs in active voice in the transcript. The numbers of transition words and summary words were identified according to the transition word list. We used the video transcription confidence score as a proxy to determine whether the users could hear the words clearly in narratives. Videos are often broken into different chunks by scenes. We used Google Video Intelligence to detect the number of scenes in the video as an indicator to determine if the video had short sections and used text processing methods to generate features from the transcript. Table 8 summarizes the features we extracted from the video content view, the methods to derive the measure, and the PEMAT criteria they fall under.

**Table 8:** Features for video understandability classification from the video content view.

Feature name	Feature description	Method	PEMAT^a^ criterion
Narrative readability	The automated readability index for narrative	Readability analysis	The material uses common everyday language
Active word count	The number of verbs in active voice in the video transcript	Syntactic analysis	The material uses active voice
Summary word count	The number of summary words in the video transcript	Semantic analysis	The material provides a summary
Transition word count	The number of transition words in the video transcript	Semantic analysis	The material presents information in a logical sequence
Video transcription confidence	The video transcription confidence score	Auto transcription	The material allows users to hear the words clearly
Text detection confidence	Text recognition confidence score	Optical character recognition	The text on the screen is easy to read
Scene count	The total number of scenes in the video	Scene detection	The material breaks or “chunks” information into short sections
Transcript word count	The total number of words in the video transcript	Auto transcription	Medical information encoded in the video
Transcript unique word	The total number of unique words in the transcript	Auto transcription	Medical information encoded in the video
Transcript sentence count	The number of unique words in a video	Auto transcription	Medical information encoded in the video
Transcript medical term	The total number of medical terms in the video narrative	Medical entity recognition	Medical information encoded in the video
Video object	The total number of unique objects in the video	Object detection	Medical information encoded in the video

^a^PEMAT: Patient Education Materials Assessment Tool.

### Evaluating the Impact of Video Understandability on User Engagement

Building on prior studies that examined collective engagement on YouTube, we identify 3 measures of user engagement: video view count, comment count, and like count, which can be obtained from publicly available YouTube metadata [57]. To assess the causal impact of understandability on user engagement, we used a method called coarsened exact matching that reduces the impact of confounding in observational causal inference. Because our data were observational, we could not conduct randomized experiments to vary the level of understandability of videos across users and assess the resultant impact on engagement. Because user engagement may be influenced by a whole host of factors external to the content of a video, we used coarsened exact matching that produces a matched sample of videos according to the covariate distributions in the treatment and control groups (ie, videos classified as understandable or not). To achieve covariate balance, we controlled for a host of heuristic measures of video quality, such as the duration of the video, a good description or a comprehensive narrative, technical quality, credentials, and the number of days since being published.

### Ethical Considerations

Our study includes an expert evaluation process, for which we obtained approval from the institutional review board (STUDY00015114: Leveraging YouTube Video Analytics for Patient Education). The evaluation process adheres to the required ethical standards for research involving human participants. Our study did not involve the use of medical records or patient information, as the evaluation was conducted with 4 medical experts to assess the proposed approach. A formal informed consent process was implemented, and the consent form is provided in Multimedia Appendix 4 for review. No personally identifiable information was collected from the participants, and all responses were securely stored in compliance with confidentiality protocols. Each expert received a compensation of US $50 for their participation, ensuring transparency and fairness in the compensation process.

## Results

### Video Understandability Classification

We collected 9873 videos using the search keywords extracted from a patient-oriented forum and reviewed by a medical expert. Among the 9873 videos, 8963 (90.78%) had descriptions, 8719 (88.31%) had narratives, and 4327 (43.83%) had text embedded in the videos. Videos with both descriptions and narratives were included in the subsequent analyses. We applied text and video analytics techniques to extract metadata view features and video content view features. Tables S1 and S2 in Multimedia Appendix 5 report the descriptive statistics of the features of all the videos in our data collection and correlations between these features. Our cotraining model initially started with 600 labeled videos for training. The model converged after 12 iterations with a confidence threshold of 0.65. In the cotraining process, 305 videos required human annotation. All the hyperparameters were selected based on empirical experiments. Multimedia Appendix 6 reports all the hyperparameters. After the 2 classifiers converged, 2921 videos were classified as low understandability and 4891 videos were classified as high understandability. A total of 907 videos remained unlabeled. When we examined these unlabeled videos, we found that a large portion of the videos contained narratives in foreign languages, while the descriptions were in English, and hence, the classifiers could not obtain consistent results. Therefore, we grouped them into low understandability.

Table 9 shows the coefficients of the logistic regression classifiers for each view. The active word count and summary count had a significant and positive impact on understandability. The transition word count in narratives was significant, but that of description was not. Transcription confidence and text detection confidence had a positive impact on video understandability. Video duration as well as medical terms count in descriptions and transcriptions negatively affected the video understandability. The readability scores of both the description and the narratives had a significant and positive impact on video understandability.

The most significant variables were consistent with PEMAT. Low understandability videos were associated with longer duration, lengthier narratives, and a larger number of medical terminologies. For model performance, we compared our predicted results for the 100 videos included in the evaluation set. Although cross-validation is commonly used in evaluating ML models, it is not feasible to collect a large repository of labeled data to evaluate cotraining models. Therefore, we adopted a holdout evaluation that was usually used for cotraining methods. We compared our model with 3 benchmark models: logistic regression, support vector machines, and random forest. To ensure a fair comparison, we carefully tuned the model hyperparameters to obtain the best performance of the benchmark models and the proposed method. For the logistic regression, we experimented with different solvers and regularization methods. Our best performance model used liblinear solver and L2 regularization. The best performance of support vector machines was achieved by radial basis function kernel and a penalty score of 0.1. The best performance of the random forest model was achieved by max_features = log_2_, and N_estimator =100. Table 10 summarizes the classification performance of our proposed method and benchmarks. Our approach achieved a weighted precision of 0.84, a weighted recall of 0.79, and an *F*_1_-score of 0.81 in classifying videos. The results showed that the cotraining method significantly improved the video understandability classification performance. The classifiers trained on 2 views “teach” each other with the additional examples whose labels are given by the other classifier or human experts and hence improve the classification performance [58].

**Table 9:** Logistic regression model summary.

Variable name		Estimate	*P* value
***F_1_*: video metadata view**			
	Has title	−0.335	.35
	Has description	−0.217	.15
	Has tags	−0.184	.18
	Description readability	*0.367* ^a^	.07
	Active word count	*0.029*	.09
	Summary word count	*0.152*	.049
	Transition word count	0.096	.10
	Video duration	−*0.071*	.09
	Description word count	0.038	.14
	Sentence count	0.157	.12
	Description unique words	0.085	.14
	Description medical term	−0.020	.07
	Constant	−0.319	.11
***F_2_*: video content view**			
	Narrative readability	*0.132*	.03
	Active word count	*0.117*	.045
	Summary word count	*0.045*	.09
	Transition word count	*0.028*	.04
	Transcription confidence	*0.021*	.04
	Shot count	−0.254	.20
	Transcript word count	−0.036	.14
	Transcript unique word	−*0.085*	.07
	Transcript sentence count	−0.074	.14
	Transcript medical term	−*0.009*	.045
	Video object	−*0.104*	.06
	Constant	−0.272	.12

^a^Italicization indicates a significance level of *P*<.10.

**Table 10:** Video understandability classification results.

	Precision	Recall	*F*_1_-score	AUC^a^
Cotraining with logistic regression	0.84	0.79	0.81	0.91
Logistic regression	0.63	0.60	0.61	0.63
Support vector machines	0.77	0.75	0.76	0.78
Random forest	0.80	0.74	0.77	0.81

^a^AUC: area under the curve.

### Impact of the Cotraining Process on Classification Performance

**Figure 4:**
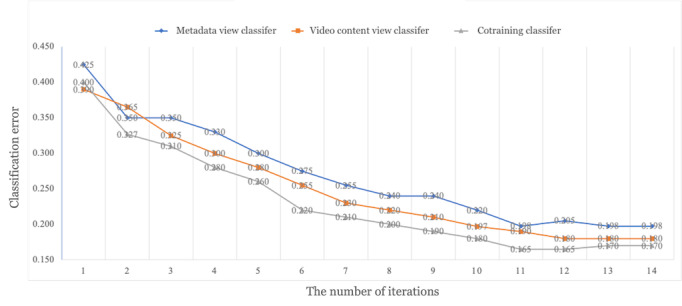
Cotraining performance by iteration.

The cotraining process combines expert efforts and ML methods to classify the video understandability according to the guidelines in the patient educational domain. One critical issue in the human-algorithm connection is to understand how this collaboration between human experts and ML algorithms improves performance. Figure 4 shows the classification error rate on the test set over iterations of training. In each iteration, new instances were added to the training process, lowering the classification error of the metadata view classifier, video content view classifier, and cotraining classifier. The reduction in classification error shows that this iterative process improves the overall performance. Furthermore, by combining human intelligence and machine intelligence from classifiers of 2 different views, the cotraining approach achieved the best performance.

### Impact of Expert Involvement on Classification Performance

The human-algorithm interaction in the cotraining process happens in two stages: (1) medical professionals provide a set of labeled examples to initialize the model training and (2) medical professionals are also involved in the cotraining process when there are inconsistent high-confidence labels predicted by 2 different classifiers. Obtaining inputs from domain experts through a human-in-the-loop algorithm design is essential to our chosen task of assessing the understandability of videos from a patient education perspective. Our design also seeks to minimize human involvement while not compromising performance. To this end, we evaluated the impact of human involvement at different stages of model learning.

**Figure 5:**
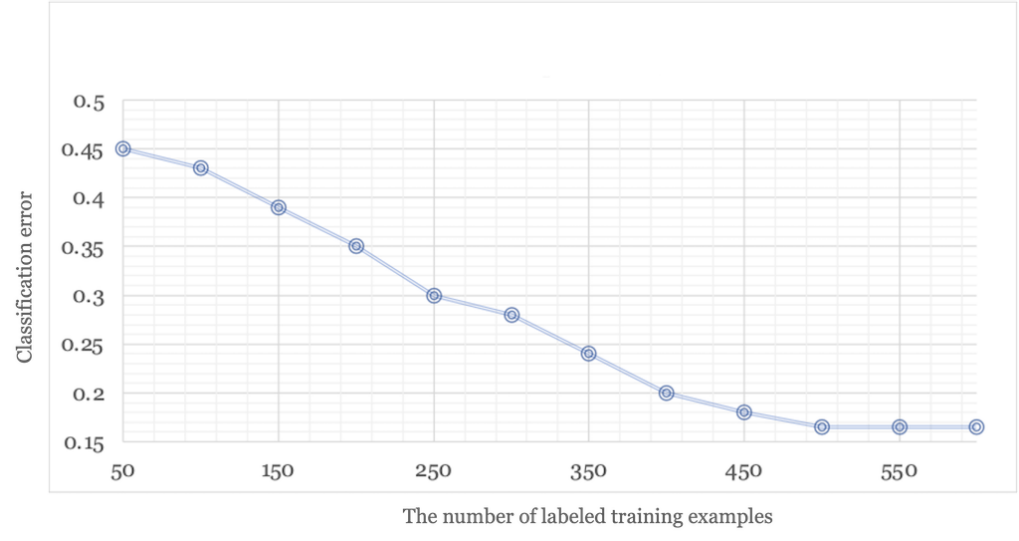
Cotraining performance versus the number of labeled training examples.

Devising a model to classify the understandability of patient educational videos requires high-quality training data. The process of creating training data involves medical professionals reviewing and categorizing videos based on guidelines. Figure 5 shows the classification error by the number of labeled training examples. As ML algorithms are dependent on the quality and quantity of their training data, we observed that increasing the number of labeled examples led to an improvement in performance. However, as for cotraining, the benefit of adding more training examples diminished when we accumulated a significant number of labeled training examples (ie, 500) for video understandability classification.

The confidence threshold determined how many predicted labels we included in the label comparison. Its purpose was to prevent the unlabeled samples from being labeled with the wrong labels, thus decreasing the ability of the learner. On the basis of the label confidence threshold, unlabeled data in each iteration was divided into 3 categories: videos with low-confidence labels, videos with consistent and high-confidence labels, and those with inconsistent but high-confidence labels. The lower the confidence threshold, the higher was the number of videos compared and evaluated in each iteration.

A lower threshold can possibly lead to a faster convergence but, at the same time, needs more human involvement during the cotraining process. A higher confidence threshold may lead to the early stopping of the training process because no new labels meet the confidence threshold. When the confidence threshold is high, the training process stops before assigning labels to all the unlabeled data. We followed the majority rule to assign the predicted labels for these unlabeled samples. We observed a negative impact on the classification performance due to early stopping from a high-confidence threshold. When the confidence threshold is too low, too many unlabeled examples are misclassified, which affects the ability of the cotraining model. As a result, we see a performance decrease when the confidence threshold is too low.

### The Importance of Understandability of Video Recommendations by Experts

We conducted a small user study to assess the impact of video understandability on experts’ decisions to recommend a YouTube video for patient education. Our automated methods reranked the search results from 20 randomly selected queries according to their video understandability classification. Four medical experts reviewed the top 10 videos according to our reranked results for each query and reported whether they would recommend the videos to patients. Precision at K is a common information retrieval measure used in modern (web-scale) information retrieval systems [59]. In web-scale retrieval, queries have thousands of relevant documents, and few users will be interested in reading all of them. Precision at K (P@K) assesses how many of the top K results are relevant (eg, P@10 or “precision at 10” corresponds to the number of relevant results among the top 10 documents). We measured the average precision at K with K varying from 1 to 10 for the 20 randomly selected queries.

**Figure 6:**
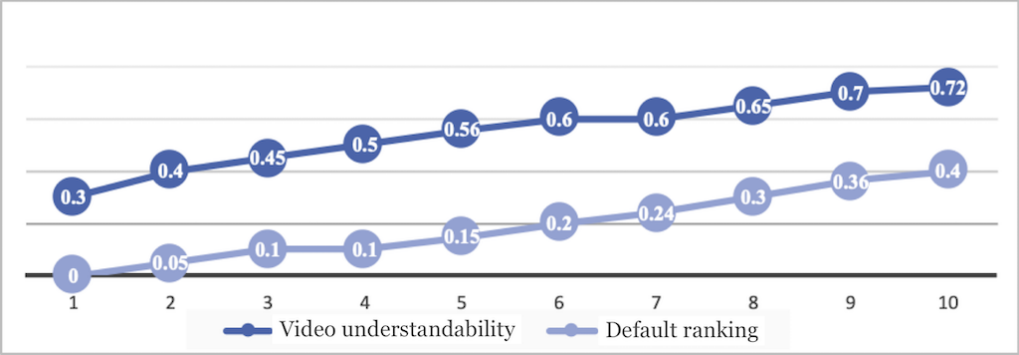
Comparison of video understandability ranking and default YouTube ranking with expert recommendation.

Figure 6 shows a chart comparing the significance of video understandability ranking. In total, 30% (6/20) of the top-ranked videos (videos ranked 1 or top 1) in understandability were recommended by an expert. None of the videos that were top ranked by YouTube’s default ranking received this recommendation. In total, 72% (144/200) of the top 10 videos were recommended by experts ranking by understandability, while only 40% (80/200) of the top 10 YouTube-ranked videos were recommended. We concluded that our understandability classification approach had considerable promise in effectively identifying patient education videos and will be evaluated further in future studies.

### Impact of Understandability on Engagement

Table 11 shows the logistic regression results for propensity score matching. After matching, there were 365 videos in the treated group and 597 videos in the control group. The summary of the balance on the entire dataset and the matched dataset is available in the Table 12. The differences in the variables between the 2 groups were significantly reduced after matching.

The matching provided a way to identify the impact of understandability on a video’s view count, like count, and comment count. The estimated treatment effect on the 3 measures of collective engagement is shown in Table 13. The results suggest that video understandability had a significant and positive impact on all 3 dimensions of engagement. Increasing the understandability of the videos can bring more viewership, more comments, and more likes. Our results highlight the importance of understandability for health care organizations and health practitioners on social media platforms.

**Table 11:** Results of the logistic regression model.

Coefficients	Estimate	*P* value
Intercept	−9.7470	<.001
Log(ChannelViewCount+1)	0.5162	<.001
Log(ChannelSubscriberCount+1)	0.2295	<.001
Log(ChannelVideoCount)	−0.7669	<.001
ContentDefinitionSD	−0.4352	<.001
Duration	0.0002	<.001
Description word count	−0.0015	.009
Description unique word	0.0069	<.001
Log(publishedDays+1)	0.2874	.002

**Table 12:** Balance on the entire dataset and matched dataset.

Variables	All data			Matched data		
	Treated, mean	Control, mean	Standardized mean difference	Treated, mean	Control, mean	Standardized mean difference
Log(channelViewCount+1)	16.0245	12.1291	1.5060	14.9886	14.9739	0.0057
Log(channelSubscriberCount+1)	10.6054	6.3278	1.3476	9.3214	9.2441	0.0243
Log(channelVideoCount+1)	5.3956	5.1962	0.1114	5.3068	5.3070	−0.0001
contentDefinitionhd	0.7960	0.5683	0.5650	0.6986	0.6986	0
contentDefinitionsd	0.2040	0.4317	−0.5650	0.3014	0.3014	0
Duration	676.7022	413.2235	0.3008	503.0027	395.4571	0.1228
word_count_description	242.9178	146.3287	0.3735	126.7836	129.3711	−0.01
unique_word_description	127.6597	76.5907	0.4522	72.8219	73.8849	−0.0094
Log(publishedDays+1)	7.4026	7.3888	0.0295	7.5097	7.5065	0.0069

**Table 13:** Estimated treatment effect.

Measure		Estimate	*P* value
**Log(view count)**			
	Intercept	8.8946	<.001
	ATE^a^	2.5523	<.001
**Log(like count)**			
	Intercept	3.2320	<.001
	ATE	2.9494	<.001
**Log(comment count)**			
	Intercept	1.6211	<.001
	ATE	3.0981	<.001

^a^ATE: average treatment effect.

## Discussion

### Principal Findings

In this study, we developed a human-in-the-loop augmented intelligence approach to assess the understandability of 9873 diabetes education videos on the YouTube social media platform in accordance with PEMAT guidelines. The cotraining classification model, which combined ML with expert input, achieved strong performance (precision=0.84, recall=0.79, and *F*_1_-score=0.81). We further examined the impact of understandability and found that higher understandability positively impacted viewer engagement (more views, likes, and comments) and increased the likelihood of expert recommendations for patient education. The findings highlight the importance of improving video understandability for enhancing patient engagement with educational materials on contextually relevant health-related topics, potentially advancing the health literacy of individuals and populations.

### Implications for Research and Practice

With complex and very large-scale data hosted by digital platforms, billions of people worldwide are accessing health care information through social media channels without any means of verifying the accuracy, understandability, relevance, and other critical criteria associated with the content being disseminated. Hence, there is an urgent need for an evidence-based approach with replicable, scalable, and generalizable AI-based methods for health literacy promotion and patient education. To the best of our knowledge, our research is the first to attempt a validated guideline-driven consolidation of rich multimedia data sources spanning video metadata and content data in text, audio, video, and structured data formats combined with a human-in-the-loop learning strategy to assess video understandability in the health care domain.

Advocates of social media in medicine highlight social media’s potential to enable patient education and empowerment [60], offering the possibility of improving health outcomes [5]. Health care organizations lack resources to create video content on the wide range of symptoms, diseases, and their progression that are treated by clinicians on a daily basis; offer easily understandable advice that can be integrated into patients’ self-care routines; or provide advice on topics that are outside the physician-patient interaction in a clinical setting. Improving clinicians’, patients’, and the public’s access to usable health information via curated and trusted video recommendations by domain experts can elevate population health literacy, empower patients, and build societal resilience.

As we demonstrate in this study, identifying, curating, and recommending relevant video materials leveraging the vast corpora of publicly available user-generated content is a feasible way to deliver personalized and contextualized health information for patient education. The adaptability of the content found on social media has enabled a variety of applications that were hitherto unthinkable. Well-designed user-generated content videos, in tandem with evidence from rigorous field experiments, could serve as part of a holistic system of care encompassing disease prevention and lifestyle changes along with resources for emotional support, better patient-physician interactions, and providing current and scientifically valid medical information to patients. The approach taken in this study, while evaluated with diabetes videos, has the potential for broader applicability across various health domains. The methods and principles developed in this research could be adapted to other chronic, acute, and infectious health conditions, such as cardiovascular disease, hypertension, pneumonia, and influenza, and broader patient education contexts, such as medication adherence and patient safety. Furthermore, the same approach can also be applied to curate videos for upskilling clinical professionals, such as surgical residents and nursing staff.

Currently, digital technologies for public health literacy and patient education are limited, lack scalability, and do not fully use the vast amount of publicly available health information found online and on social media platforms. Providing a strong open platform will provide a credible alternative to the vested interests of private organizations with proprietary technologies, which will lead to future innovations in novel data collection devices, digital platforms, and technologies in the context of health literacy initiatives.

Our methodology to develop a patient educational video system for understandability by integrating human efforts, that is, the perspectives of clinical practitioners and health care consumers, with ML algorithms is an innovative approach to a societally challenging problem. Patient empowerment and engagement are essential for appropriate disease management. For health organizations that are producing patient educational materials, our approach could be used as an educational tool for enhancing understandability in patient educational video content design. When designing educational materials, the insights from feature analysis of the ML algorithms have the potential to provide best practice guidelines regarding how organizations should engage health consumers with educational videos for varying levels of health literacy. Understandability can be further improved with the use of visual aids, summaries, and tangible tools such as personalized charts. Our study could add to patient communication and education literature and practice by enabling clinical practitioners to identify the most understandable, medically informative, and engaging videos for their patients as digital therapy. By combining algorithmic approaches with impact evaluation, this approach seeks to identify effective intervention methods that enable platform designers and clinicians to retrieve the most appropriate videos as digital therapeutic tools. Our approach can be extended to incorporate recent efforts by digital platforms and reputed national and international health organizations to identify authoritative sources of health information on social media channels and amplify credible content [23].

### Limitations and Future Directions

This study has some limitations. Our study is built on the PEMAT guidelines developed by AHRQ. Although it is the most prevalent evaluation tool on patient education materials, PEMAT is not designed for user-generated content but for materials produced by health care organizations. The PEMAT criteria may require adaptation or extension to YouTube videos in evaluating subcriteria, such as whether the materials used for illustration were uncluttered, the technical quality of the video was satisfactory, and so on. In future work, we would like to explore alternative assessment tools or develop one that is more suitable for user-generated videos.

We also relied heavily on the evaluation of patient education materials from 4 physician evaluators, which poses the risk of evaluator bias. The calculated κ score indicates that there was variability in the reviewers’ use of the tools. However, we minimized this limitation by using the adjudication process for each item with a discrepancy, which is the accepted method to achieve consensus scores [61]. Additional video features that focus on esthetics, production qualities, whether the video contains a human, and so on were not used in this study due to our restricted definition of video understandability following the PEMAT guidelines. In addition to patient educational guidelines, it may also be necessary to examine factors such as concordance, which is the similarity or shared identity between physicians and patients based on a demographic attribute, such as race, gender, ethnicity, or age [62]. While understandability of content is an important criterion, other requirements such as accuracy of content, inclusivity and representativeness of content and its narrators, credibility and trustworthiness of the sources producing the videos, and others are equally critical. Finally, the challenges associated with the logistics (such as when, how, who will, and who to) of the delivery of the video recommendations have to be investigated using rigorous implementation science theories and frameworks. Future work will address these issues to potentially improve the reach and educational value of the recommended videos.

### Conclusions

This study makes two contributions to the literature in the multidisciplinary area of digital therapeutics for health literacy and human-algorithm collaboration. The first contribution is the development of a human-in-the-loop augmented intelligence method that incorporates human judgment and expertise into an ML-driven computational approach that characterizes the search for cognitively demanding information on social media by combining human cognitive capabilities and AI systems. Our approach uses inputs from domain experts and PEMAT-based patient education constructs combined with ML and NLP methods to design and implement an automated tool that analyzes the understandability of YouTube videos from the perspective of patient education. The second contribution is to enable a better understanding of how patients assimilate health care information by assessing the impact of video understandability on viewer engagement with the videos. Our proposed solution can also provide health organizations with actionable guidance in designing and creating patient educational videos. Our findings can offer policy implications for the design and deployment of such digital artifacts for health literacy improvement, use of health care resources, and the quality of delivered care.

Future work can build on our approach to create a method of automated video retrieval that will accommodate viewers’ varying levels of both health and functional literacy and engagement. Future work can also build on the methods developed in this paper to develop multicriteria recommendations for a range of video content on topics such as education, climate change, financial literacy, and virtual communities based on metadata and video features from large social media platforms, such as YouTube.

## Appendix

Multimedia Appendix 1 Search terms for creating a corpus of videos.

Multimedia Appendix 2 Video attributes collected using YouTube data application programming interface.

Multimedia Appendix 3 Medical entity extraction performance.

Multimedia Appendix 4 Consent form.

Multimedia Appendix 5 Descriptive statistics of features for video understandability classification.

Multimedia Appendix 6 Performance metrics and hyperparameters of the proposed approach.

## Abbreviations

AHRQ: Agency for Healthcare Research and Quality
AI: artificial intelligence
API: application programming interface
CDC: Centers for Disease Control and Prevention
ML: machine learning
N/A: not applicable
NLP: natural language processing
OCR: optical character recognition
PEMAT: Patient Education Materials Assessment Tool

## Data Availability

The datasets generated or analyzed during this study are available from the corresponding author on reasonable request.
