# Supplementary material for: Promoting Health Literacy With Human-in-the-Loop Video Understandability Classification of YouTube Videos: Development and Evaluation Study
Source: J Med Internet Res. 2025 Apr 8;27:e56080. doi: 10.2196/56080 (PMC11984000; doi:10.2196/56080)
Supplement: Multimedia Appendix 1 [file jmir_v27i1e56080_app1.docx]

**Multimedia Appendix 1. Search Terms for Creating a Corpus of Videos**

We collected 9,873 unique videos using 235 search terms, which serve as the data for the current study on video understandability. These terms were collected with help from physicians and diabetes educators. These terms appear below:

**Table A1. Video Search Keywords**

| Diabetes | insulin resistance | HemoglobinA1c | insulin infusion pump | Toujeo |
| --- | --- | --- | --- | --- |
| diabetes causes | insulin resistance syndrome | high glucose | insulin injection | Tradjenta |
| diabetes complication | insulin secretion | iv glucose tolerance test | insulin needles | Tresiba |
| diabetes cure | insulin sensitive | lifescan | insulin pen injector | Welchol |
| diabetes depression | islet amyloid polypeptide | normal blood glucose levels | insulin pump | artificial sweetener |
| diabetes diagnosis | Islet cell antibodies | normal glucose tolerance | insulin syringe | diabetes diet |
| diabetes exercise | islets of Langerhans | oral glucose challenge | insulin therapy | diabetes prevention |
| diabetes high risk | lipodystrophy | Oral glucose tolerance test | intermediate acting insulin | diabetes prevention program |
| diabetes information | obesity | plasma concentration | Invokana | diabetes target range |
| diabetes medication | pancreas | plasma glucose | islet cell transplantation | diabetic diet |
| Diabetes Mellitus | pancreatic exocrine disease | post glucose tolerance test | Januvia | glycemic index |
| diabetes self-care | pancreatic islet | post prandial glucose | Lantus | physical activity |
| diabetes self-management | reduced insulin | Post-prandial glucose | Levemir | adult blindness |
| diabetes supplies | syndrome X | PPG | linagliptin | Autonomic Neuropathy |
| diabetes surgery | Acanthosis nigricans | urine glucose | Lispro | cellulitis |
| diabetes symptoms | body mass index | Acarbose | long acting insulin | diabetes and anorexia |
| diabetes testing | body weight | ACTOS | meglitinides | diabetes and female sexual dysfunction |
| diabetes treatment | diabetes risk factor | alogliptin | metformin | diabetes and nausea |
| diabetes type 1 symptoms | diabetes risk factors | alpha-glucosidase inhibitors | miglitol | diabetes and reduced sexual desire women |
| diabetes type 2 symptoms | glycosuria | Apidra | Nateglinide | diabetes and vomiting |
| diets for diabetes | hirsutism | artificial pancreas | Nesina | diabetes infections |
| DM | hyperandrogenism | Aspart | Novolin | diabetes myocardial infarct |
| Gestational diabetes | increased urination | Avandia | Novolin N | diabetes staph infection |
| gestational DM | increased water intake | bariatric surgery | Novolog | diabetes urinary tract infections |
| increased risk of diabetes | intermediate hyperglycemia | Biguanides | NPH insulin | diabetes UTI |
| insulin dependent diabetes mellitus | PCOS | bolus insulin | Onglyza | diabetic coma |
| juvenile diabetes | polycystic ovarian syndrome | Canagliflozin | oral hypoglycemic agents | diabetic dermopathy |
| Type 1 | polydipsia | carbohydrate counting | pancreatic transplantation | diabetic foot |
| Type 1 diabetes | polyuria | colesevelam | parenteral glucagon emergency | diabetic heart disease |
| type 1 DM | weight loss | Cycloset | pioglitazone | diabetic keto acidosis |
| Type 2 | weight gain | Detemir | Pramlintide | diabetic mononeuropathy |
| Type 2 diabetes | blood glucose | diabetes clinical trails | prandin | diabetic nephropathy |
| Type 2 DM | blood pressure | Diabetes medicine | precose | diabetic neuropathy |
| type I diabetes | continuous glucose monitoring systems | DPP-4 Inhibitors | rapid acting insulin | diabetic peripheral vascular disease |
| type I DM | c-peptide test | FlexPen | Regular insulin | diabetic polyneuropathy |
| Type II | fasting glucose | Glargine | Repaglinide | diabetic retinopathy |
| Type II diabetes | fasting plasma glucose | Glucophage | Rosiglitazone | diabetic skin spots |
| Type II DM | fingerstick glucose test | Glulisine | Ryzodeg | End stage renal disease |
| foot ulcers | FPG | Glumetza | saxagliptin | ESRD |
| diabetes lose weight | glucometers | glyset | SGLT2 inhibitors | foot ulcers |
| borderline diabetes | glucose meter | Humalog | short acting insulin | gangrene |
| diabetes kit | glucose monitor | Humulin | Sitagliptin | gastroparesis |
| onset diabetes | glucose test strip | Humulin N | starlix | hypoglycemia |
| diabetes magazines | glucose tolerance test | inhaled insulin | SymlinPen | insulin reaction |
| herbal treatment for diabetes | HbA1c | injection site rotation | thiazolidinediones | insulin shock |
| neuropathy | peripheral neuropathy | Toujeo | Tradjenta | hyperglycemia |
| Tresiba | artificial sweetener | diabetes diet | hypoglycemia | Welchol |
