# Supplementary material for: Promoting Health Literacy With Human-in-the-Loop Video Understandability Classification of YouTube Videos: Development and Evaluation Study
Source: J Med Internet Res. 2025 Apr 8;27:e56080. doi: 10.2196/56080 (PMC11984000; doi:10.2196/56080)
Supplement: Multimedia Appendix 2 [file jmir_v27i1e56080_app2.docx]

**Multimedia Appendix 2. Video Attributes Collected using YouTube Data API**

We collected the top 50 videos for each search term with YouTube Data API and stored the videos, their rankings, and metadata in a database for further analysis. The attributes we collected from each video are grouped and illustrated in Figure A1 below. Attributes related to video snippet and content details are generated at the time of video upload while video usage is generated by user engagement over time and the statistics are from the day of video data collection.

|  |
| --- |
| Figure A1: Data Attributes from YouTube Data API |
