# Supplementary material for: Promoting Health Literacy With Human-in-the-Loop Video Understandability Classification of YouTube Videos: Development and Evaluation Study
Source: J Med Internet Res. 2025 Apr 8;27:e56080. doi: 10.2196/56080 (PMC11984000; doi:10.2196/56080)
Supplement: Multimedia Appendix 3 [file jmir_v27i1e56080_app3.docx]

**Multimedia Appendix 3. Medical Entity Extraction Performance**

We implement a Bidirectional Long Short-term Memory (LSTM) model to extract medical terms from the user-generated video descriptions at sentence level. The video descriptions are first parsed into individual sentences. The model consists of two LSTMs that run in parallel: one on the input sequence and the other on the reverse of the input sequence. At each time step, the hidden state of the BiLSTM is the concatenation of the forward and backward hidden states. This setup allows the hidden state to capture both the past and the future information. To reduce computational complexity, we train a 300-dimensional Glove embedding model, meaning each word is converted to a 300-dimensional semantic vector. Then the word sequence is represented as an embedding sequence, which is passed to the BiLSTM layer. Instead of using a large hidden layer size, we use 150 neurons in the BiLSTM layer to avoid over-fitting. This hidden layer size setup has also been successfully tested in other studies. The outputs of the BiLSTM layers are then processed by a CRF classifier, which predicts the semantic type of each word in the input sentence. The BiLSTM model was trained on 4,000 annotated sentences, with 1,000 sentences as the validation set (for cross validation). Another 1,000 annotated sentences were used as the test set. Table A1 below shows the performance of the BiLSTM model by category.

**Table A2. Medical Term Extraction Performance By Category**

| **Semantic Group** | **MetaMap+ CHV** | | | **CRF** | | | **BiLSTM RNN** | | |
| --- | --- | --- | --- | --- | --- | --- | --- | --- | --- |
|  | **P** | **R** | **F** | **P** | **R** | **F** | **P** | **R** | **F** |
| All | 58.94% | 36.28% | 44.19% | **97.32%** | 60.09% | 73.29% | 87.43% | **87.81%** | **87.32%** |
| Body part | 75.40% | 55.30% | 63.80% | **95.20%** | 67.00% | 78.60% | 93.90% | **89.80%** | **91.80%** |
| Chemicals or Drugs | 67.80% | 35.20% | 46.34% | **98.90%** | 65.90% | 79.10% | 82.10% | **91.50%** | **86.50%** |
| Medical devices | 14.90% | 20.70% | 17.33% | **99.10%** | 79.00% | 87.90% | 94.40% | **91.90%** | **93.10%** |
| Medical events | 65.60% | 45.60% | 53.80% | **99.50%** | 38.60% | 55.60% | 91.90% | **77.30%** | **84.00%** |
| Medical professionals | 57.90% | 12.30% | 20.29% | **99.80%** | 91.10% | 95.30% | 98.60% | **96.70%** | **97.60%** |
| Medical procedures | 12.00% | 13.00% | 12.48% | 70.30% | 31.80% | 43.80% | **88.20%** | **82.50%** | **85.30%** |
