# Supplementary material for: Promoting Health Literacy With Human-in-the-Loop Video Understandability Classification of YouTube Videos: Development and Evaluation Study
Source: J Med Internet Res. 2025 Apr 8;27:e56080. doi: 10.2196/56080 (PMC11984000; doi:10.2196/56080)
Supplement: Multimedia Appendix 4 [file jmir_v27i1e56080_app4.docx]

**Multimedia Appendix 4. Consent Form**

Our study went through the IRB review process across all authors’ academic institutes. Below please see the consent form for participants in one of the institutes. Phone numbers and emails of the research team member in the form have been removed to maintain anonymity.

| Consent Form This assessment of YouTube videos on diabetes using criteria such as understandability is part of a research study funded by the National Institutes of Health (NIH) and conducted by Prof. Rema Padman at Carnegie Mellon University in collaboration with researchers at Michigan State University and Arizona State University. The purpose of the research is to apply validated criteria for identifying and retrieving curated, medically informative YouTube videos on diabetes for patient education. Procedures After completing this consent form, Aim 1 participants will be introduced to a list of 200 short duration videos (< 6 minutes each) with links to the actual videos. Participants will be asked to watch and rate the videos on pre-specified criteria and also answer whether the participant will recommend video for patient education. We anticipate that this task will consume approximately 20 hours in total and will need to be completed within one month from the start of the assignment. Participant Requirements Participation in this study is limited to individuals age 21 and older and will comprise both graduate students with and without medical knowledge and clinicians from the researchers’ networks. Voluntary ParticipationYour participation in this research is voluntary. You may discontinue participation at any time during the research activity. You may print a copy of this consent form for your records.Risks The risks and discomfort associated with participation in this study are no greater than those ordinarily encountered in daily life or during other online activities. The privacy of the study participants is protected and personal identifiers used for recruiting will not be stored in the same file as the data collected during the experiment. However, users may experience some discomfort while watching some of the videos on diabetes details and there is a potential risk of breach of confidentiality. Benefits Participants will receive no direct benefit by participating. Compensation & Costs Participants in this study will receive a compensation of $50 each after completing the assessment survey. There will be no cost for participating in this study. There will be no partial payment if the participant completes only part of the study.  Confidentiality  By participating in this research, participants understand and agree that Carnegie Mellon may be required to disclose their consent form, data and other personally identifiable information as required by law, regulation, subpoena or court order. The sponsor, NIH, has the right to access the research records. Individuals with oversight of research responsibilities may also have access to the research records.  Otherwise, participant confidentiality will be maintained by keeping participant data and consent form separate. The consent form will be stored in a secure location on a secure server and will not be disclosed to third parties. Sharing of data with other researchers will only be done in such a manner that participants will not be identified. By participating, participants understand and agree that the data and information gathered during this study may be used by the researchers and published and/or disclosed to others outside of the participating universities. However, participant name, contact information and other direct personal identifiers will not be mentioned in any such publication or dissemination of the research data and/or results by the researchers. Note that per regulation all research data must be kept for a minimum of 3 years.  This research is covered by a Certificate of Confidentiality from the National Institutes of Health. The researchers with this Certificate may not disclose or use information or documents that may identify you in any federal, state, or local civil, criminal, administrative, legislative, or other action, suit, or proceeding, or be used as evidence, for example, if there is a court subpoena, unless you have consented for this use. Information or documents protected by this Certificate cannot be disclosed to anyone else who is not connected with the research except, if there is a federal, state, or local law that requires disclosure (such as to report child abuse or communicable diseases but not for federal, state, or local civil, criminal, administrative, legislative, or other proceedings, see below); if you have consented to the disclosure, including for your medical treatment; or if it is used for other scientific research, as allowed by federal regulations protecting research subjects.  The Certificate cannot be used to refuse a request for information from personnel of the United States federal or state government agency sponsoring the project that is needed for auditing or program evaluation by National Institutes of Health which is funding this project or for information that must be disclosed in order to meet the requirements of the federal Food and Drug Administration (FDA). You should understand that a Certificate of Confidentiality does not prevent you from voluntarily releasing information about yourself or your involvement in this research. If you want your research information released to an insurer, medical care provider, or any other person not connected with the research, you must provide consent to allow the researchers to release it.  Right to Ask Questions & Contact Information  If participants have any questions about this study, feel free to contact: Dr. Rema Padman  Address: Heinz College of Information Systems and Public Policy, CMU; 5000 Forbes Avenue, PA 15213; Phone 412 XXX-XXX; Email [XXX@cmu.edu](mailto:XXX@cmu.edu)  If participants have questions later, desire additional information, or wish to withdraw participation, please contact the research team member by mail, phone or e-mail in accordance with the contact information listed above. If participants have questions pertaining to their rights as a research participant or to report concerns about this study, they should contact the Office of Research Integrity and Compliance at Carnegie Mellon University.  Email: irb-review@andrew.cmu.edu .  Phone: 412-XXX-XXXX.  Checkbox: "I am age 21 years or older."  Checkbox: "I have read and understand the information above."  Checkbox: "I want to participate in this research and continue with the evaluation." |
| --- |
