# Supplementary material for: Promoting Health Literacy With Human-in-the-Loop Video Understandability Classification of YouTube Videos: Development and Evaluation Study
Source: J Med Internet Res. 2025 Apr 8;27:e56080. doi: 10.2196/56080 (PMC11984000; doi:10.2196/56080)
Supplement: Multimedia Appendix 5 [file jmir_v27i1e56080_app5.docx]

**Multimedia Appendix 5. Descriptive Statistics of Features for Video Understandability Classification**

In total, we collected 9,873 videos using the search keywords identified by a medical expert. Among these videos, 8,963 videos have descriptions, 8,719 have narratives, 4,327 of them have text embedded in the videos. We applied text and video analytics techniques. Table A3 reports the descriptive statistics of the features of all the videos in our data collection.

**Table A3. Descriptive Statistics of Features for Video Understandability Classification**

| **View** | **Variable Name** | **N** | **Yes** | | | **No** | | |
| --- | --- | --- | --- | --- | --- | --- | --- | --- |
| Video meta data | Has title | 9873 | 9873 | | | 0 | | |
|  | Has description | 9873 | 8963 | | | 910 | | |
|  | Has tags | 9873 | 9873 | | | 0 | | |
|  |  |  | **Min** | **Q1** | **Mean** | **Median** | **Q3** | **Max** |
|  | Description readability | 8963 | 0 | 6.6 | 9.9 | 9.4 | 13.1 | 18 |
|  | Active verb count | 8963 | 0 | 1 | 16.7 | 4 | 18 | 170 |
|  | Summary word count | 8963 | 0 | 0 | 0.02 | 0 | 0 | 11 |
|  | Transition word count | 8963 | 0 | 0 | 2.5 | 1 | 3 | 39 |
|  | Video duration | 9873 | 1 | 67 | 387.6 | 168 | 388 | 26156 |
|  | Description word count | 8963 | 1 | 18 | 153.8 | 58 | 197 | 1118 |
|  | Sentence count | 8963 | 1 | 1 | 6.9 | 2 | 7 | 61 |
|  | Description unique word count | 8963 | 1 | 11 | 126.2 | 38 | 108 | 388 |
|  | Description medical term Count | 8963 | 0 | 0 | 3.7 | 3 | 7 | 125 |
| Video content | Narrative readability | 8719 | 0 | 7.8 | 10.5 | 10 | 14 | 19 |
|  | Active verb count | 8719 | 0 | 1 | 20.2 | 6 | 25 | 205 |
|  | Summary word count | 8719 | 0 | 0 | 0.1 | 0 | 2 | 25 |
|  | Transition word count | 8719 | 0 | 0 | 3.5 | 2 | 5 | 47 |
|  | Transcription confidence | 8719 | 0 | 0.37 | 0.73 | 0.65 | 0.84 | 0.99 |
|  | Text detection confidence | 4327 | 0 | 0.24 | 0.63 | 0.57 | 0.74 | 0.99 |
|  | Shot count | 9873 | 1 | 4 | 12.6 | 11 | 28 | 141 |
|  | Transcript word count | 8719 | 1 | 131 | 526 | 312 | 754 | 49312 |
|  | Transcript unique word count | 8719 | 1 | 26 | 215.7 | 107 | 389 | 614 |
|  | Transcript sentence count | 8719 | 1 | 6 | 32.6 | 24 | 67 | 4123 |
|  | Transcript medical term count | 8719 | 0 | 5 | 17.4 | 13 | 35 | 135 |
|  | Video object count | 9873 | 1 | 11 | 43.6 | 38 | 79 | 127 |

We examined the correlations of the numeric variables among the videos with descriptions, narratives, and text on the screen. The correlation analysis results are reported in Table A4.

**Table A4. Correlation Analysis of Features for Video Understandability Classification**
