# Supplementary material for: Promoting Health Literacy With Human-in-the-Loop Video Understandability Classification of YouTube Videos: Development and Evaluation Study
Source: J Med Internet Res. 2025 Apr 8;27:e56080. doi: 10.2196/56080 (PMC11984000; doi:10.2196/56080)
Supplement: Multimedia Appendix 6 [file jmir_v27i1e56080_app6.docx]

**Multimedia Appendix 6. Performance Metrics and Hyperparameters of the Proposed Approach**

In this study, we adopt common classification performance evaluation metrics, precision, recall, F1, ROC Curve and AUC score, to assess the performance of our approach. Precision measures the accuracy of the positive predictions made by the model. It is computed as the proportion of the instances predicted as positive that were actually correct. Precision is calculated with the following equation:

Precision = $\frac{True Positives}{True Positives+False Positives}$

- **True Positives (TP)**: The model correctly predicts a positive class.
- **False Positives (FP)**: The model incorrectly predicts a positive class.

Recall measures how well the model can identify all relevant instances of the positive class. It computes the proportion of actual positives that were correctly identified by the model. Recall is calculated with the following equation:

Recall = $\frac{True Positives}{True Positives+False Negatives}$

- **False Negatives (FN)**: The model fails to predict a positive class when it should have.

The F1 score is the harmonic mean of precision and recall, providing a single metric that balances both. F1 score is computed with the following equation:

F1 = $2\times\frac{Precision \times Recall}{Precision+Recall}$

The Receiver Operating Characteristic (ROC) curve is a graphical representation of a classifier’s performance by plotting the True Positive Rate (Recall) against the False Positive Rate (FPR) at different classification thresholds. **AUC** stands for **Area Under the Curve**. The AUC score represents the area under the ROC curve and summarizes the overall ability of the model to distinguish between the positive and negative classes. It provides a single scalar value that quantifies the classifier’s performance across all thresholds.

Our proposed approach leverages a logistic regression classification model in the co-training framework. All the hyperparameters of our proposed approach are reported in the Table A5 below.

**Table A5. Hyperparameters of Our Proposed Approach**

|  | Hyperparameter | Value |
| --- | --- | --- |
| Logistic Regression Classification | Solver | Liblinear Solver |
|  | Regularization | L2 Regularization |
| Co-training | Confidence threshold | 0.65 |
